# Electrosensory neural responses to natural electro-communication stimuli are distributed along a continuum

**DOI:** 10.1371/journal.pone.0175322

**Published:** 2017-04-06

**Authors:** Michael K. J. Sproule, Maurice J. Chacron

**Affiliations:** Department of Physiology, McGill University, Montreal, Québec, Canada; University of Sussex, UNITED KINGDOM

## Abstract

Neural heterogeneities are seen ubiquitously within the brain and greatly complicate classification efforts. Here we tested whether the responses of an anatomically well-characterized sensory neuron population to natural stimuli could be used for functional classification. To do so, we recorded from pyramidal cells within the electrosensory lateral line lobe (ELL) of the weakly electric fish *Apteronotus leptorhynchus* in response to natural electro-communication stimuli as these cells can be anatomically classified into six different types. We then used two independent methodologies to functionally classify responses: one relies of reducing the dimensionality of a feature space while the other directly compares the responses themselves. Both methodologies gave rise to qualitatively similar results: while ON and OFF-type cells could easily be distinguished from one another, ELL pyramidal neuron responses are actually distributed along a continuum rather than forming distinct clusters due to heterogeneities. We discuss the implications of our results for neural coding and highlight some potential advantages.

## Introduction

Understanding the neural code remains a central problem in neuroscience and is, in part, complicated by the fact that neurons, even within the same type, display strong heterogeneities [1–4]. Such heterogeneities can arise because of anatomical [5–7], molecular [8–11], or electrophysiological [12] differences. However, mappings between neural classifications made using each category have proven difficult to obtain [13], which is in part due to differences that are not taken into account (e.g., morphological, intrinsic firing, or synaptic connections) and the fact that neurons with vastly different molecular attributes can display similar electrophysiological properties [9, 14, 15]. It has been proposed that classifications based on neuronal function [16, 17] could help explain neural heterogeneities and provide critical insight into the neural code [18]. Here we tested whether the responses of electrosensory pyramidal neurons to natural electrosensory stimuli could be functionally classified based on their responses to stimuli alone.

Gymnotiform wave-type weakly electric fish offer an attractive system to investigate functional classifications of neural responses because of well-characterized neural circuits at the anatomical, molecular, and electrophysiological levels [19–24]. These fish generate a quasi-sinusoidal electric field around their body through the electric organ discharge (EOD). They sense amplitude modulations of this field through an array of electroreceptors scattered on their skin surface that make synaptic contact onto pyramidal cells within the electrosensory lateral line lobe (ELL) [25]. Pyramidal cells display strong heterogeneities and their anatomical, morphological, molecular, and electrophysiological attributes have been well characterized [19, 24]. Pyramidal cells can be anatomically classified into two categories based on the presence or absence of basilar dendrites. This anatomical classification can be directly mapped into a functional classification, as basilar pyramidal cells respond to increases in EOD amplitude (i.e., are On-type) whereas non-basilar pyramidal cells instead respond to decreases in EOD amplitude (i.e., are Off-type) [26–28]. Studies have furthermore shown that the ELL is organized into columns each consisting of six pyramidal cell anatomical classes (basilar and non-basilar deep, intermediate, and superficial), with each column receiving identical electroreceptor input [19]. Superficial pyramidal cell somata can be found most superficially within the pyramidal cell layer. These cells exhibit the largest apical dendritic trees, receive large amounts of feedback, and respond most selectively to electrosensory stimuli. In contrast, deep pyramidal cell somata are found deep within the pyramidal cell layer. These cells instead possess the smallest apical dendritic trees, receive the least amount of feedback, and display responses to electrosensory stimuli that are reminiscent of those of electroreceptors [19, 22, 24]. As the name implies, intermediate pyramidal cells have attributes that lie in between the deep and superficial extremes. There exists a mapping between morphological and electrophysiological properties. Indeed, previous studies have found a strong negative correlation (-0.8) between apical dendritic length and the baseline (i.e., in the absence of stimulation but in the presence of the animal’s unmodulated EOD) firing rate (S1 Fig) [29, 30]. All six pyramidal cell anatomical classes project to higher brain structures [28, 29].

When two conspecifics come into close proximity (<1 m), interference between their EODs gives rise to a beat that consists, in part, of a sinusoidal amplitude modulation. Subsequently, fish can then emit communication calls that consist of transient (<100 ms) increases in EOD frequency [31]. Such “chirps” always occur on top of the beat and give rise to appropriate behavioral responses [32, 33]. ELL pyramidal cell responses to chirps have been well documented [21, 32, 34–36]. Here we tested whether these responses could be used to functionally classify ELL pyramidal cells.

## Methods

### Animal husbandry

Adult specimens of either sex of the weakly electric fish *Apteronotus leptorhynchus* were obtained from local tropical fish suppliers and acclimated to housing tanks for a period of 2 weeks prior to experimentation according to published guidelines [37]. Fish were housed in 60-gallon tanks with ample number of shelters for up to 10 individuals, were fed once daily a diet comprising of brine shrimp, bloodworms, or daphnia, and were housed under conditions of near constant darkness. Tank water was made up by adding stock solution containing 20 g/L MgSO4.7H2O, 8 g/L KCl, 2.2 g NaSO4, and 126 g/L CaSO4.2H2O to distilled water to achieve a final conductivity of ~ 800 μS/cm. All chemicals were obtained from Sigma-Aldrich. pH was maintained between 7.1 and 7.3 while the temperature was held between 27 and 30°C. All animal procedures were approved by McGill University’s animal care committee.

### Surgery

Surgeries were performed on animals within the experimental tank which was first filled with water familiar to the animal and heated to ~27°C as done previously [4, 38–43]. Upon transferal from their housing tank to the experimental tank animals were paralyzed through an intramuscular injection of tubocurarine chloride hydrate (150 μL at a concentration of 2.5 mM). Fish were then respirated with aerated tank water flowing across the animals’ gills at a constant rate of 10mL/min. The skin covering the skull surface to be exposed and the immediately surrounding area was anesthetized with a topical application of 2% lidocaine. Using a scalpel, skin was subsequently removed from the skull above the hindbrain contralateral to the side of the fish to be stimulated during recordings. The animal was then glued to a metal post via a portion of exposed skull anterior to the recording site in order to stabilize its position in space. Using a surgical drill a small window ~5mm^2^ was made over the hindbrain.

### Stimulation

The electric organ (EO) of *A*. *leptorhynchus* is neurogenic. As such, the animal’s electric field is unaffected by curare-like drugs. Amplitude modulations of the animals own EOD were obtained in the following way. A function generator was triggered to output one cycle of a sinewave on each EOD cycle. The frequency of the sinewave was set slightly (~20–30 Hz) higher than the EOD frequency, thereby generating a quasi-sinusoidal waveform that is phase-locked to the animal’s own EOD. This waveform signal was then multiplied (MT3 multiplier, Tucker Davis Technologies) by an amplitude modulation waveform (i.e. the stimulus). The resulting signal was then isolated from ground (A395 linear stimulus isolator, World Precision Instruments) before being applied to the experimental tank via two chloridized silver wire electrodes (~ 30cm separation) located on either side of the animal. The contrast was ~20%. The naturalistic stimuli employed in the current study consisted of amplitude modulations resulting from four agonistic communication signals with duration 14 ms and consisting of a 60 Hz increase in frequency that occurred at different phases of a sinusoidal background signal with frequency 5 Hz. Two On-type chirps occurring at beat phases π and 3π/2 as well as 2 Off-type chirps occurring at 0 and π/2 were utilized in this study. Each stimulus was ~23 sec in duration and was composed of the 5 Hz beat with the first chirp trial occurring at 0.9 sec and each of the subsequent 19 trials occurring every 1.1 sec afterward.

### Recordings

Extracellular recordings (n = 90) were made from pyramidal cells within the ELL lateral segment (LS) using metal-filled micropipettes [44]. Recordings were targeted exclusively to the LS because pyramidal cells within this segment display the strongest response to chirp stimuli [35]. Recordings were amplified using an A-M systems 1700 amplifier before being digitized, by a Power1401 operating Spike2 software (Cambridge Electronic Design, Cambridge, UK), at a sampling rate of 10 kHz.

### Preprocessing

**Fig 1:**
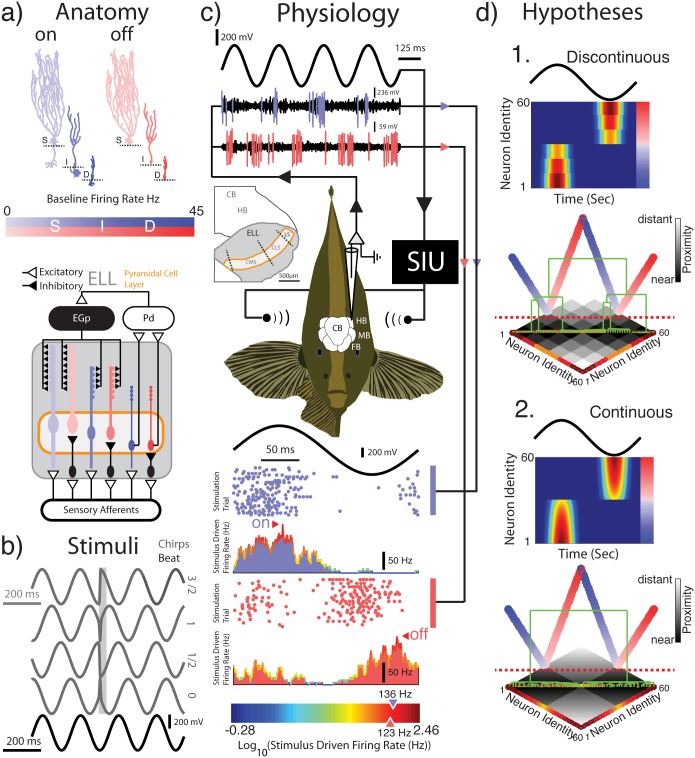
Establishing a functional classification using naturalistic communication stimuli. **A:** There are two types of pyramidal neurons, On- (blue) and Off- (red) type, which can be distinguished anatomically by the presence and absence of basilar dendrites, respectively (top). On- and Off-type pyramidal cells can furthermore be subdivided into six classes: On and Off-type superficial (S) intermediate (I) and deep (D) types which each exhibits different sized apical dendritic trees. There is a strong negative correlation between the size of the apical dendritic tree and the baseline (i.e., in the absence of stimulation) firing rate (S1 Fig). The baseline firing rate is indicated by colour saturation as per the colour bar above the circuit diagram. At the circuit level (bottom) within the pyramidal cell layer (orange boarder) all neurons receive input from sensory afferents encoding the animal’s self-generated electric field. On-type cells receive direct inputs from these afferents whereas Off-type cells receive indirect input via local inhibitory interneurons. All neuron classes project to the midbrain torus Semicircularis (not pictured here) while only deep neurons project to praeminentialis dorsalis (Pd) which provides different degrees of inhibitory feedback to superficial and intermediate pyramidal neurons via the eminentia granularis pars posterior (EGP). **B:** The four chirp stimuli featured in this study are shown in dark grey. A 25 ms response window following chirp onset is also indicated by a light grey window for two On-type chirps (3π/2, π) and the two Off-type chirps (π/2, 0). The 5 Hz beat stimulus is shown in black. **C:** A stimulus waveform is played to an awake and behaving animal while recordings are obtained from pyramidal cells within the lateral segment (LS) of the ELL. Example recordings from one On-type and one Off-type neuron are shown in response to a 5 Hz beat. Spike waveforms identified using spike sorting software are indicated for each cell (blue and red). The spike times were used to generate raster plots and peristimulus time histograms (as seen below the experimental setup). Example cells have peak stimulus driven firing rates of 136 Hz (On-type) and 123 Hz (Off-type) and their responses to the beat are in anti-phase. The color gradient in the color bar (bottom) is indicative of the response magnitude of recorded units (i.e. On- or Off-type). The transition from blue to red reflects an increase in response magnitude as the logarithm in base 10 of the stimulus driven peak-firing rate. **D:** A priori it is unclear whether ELL pyramidal cells can be functionally classified based on their responses to natural communication signals alone. There are two hypotheses: 1. Responses form distinct clusters This is schematized by a heatmap of response magnitude showing distinct response profiles. Directly beneath a hierarchical agglomerative clustering algorithm applied to a pairwise distance matrix representing the above heatmap results in a dendrogram (green) which is clearly divisible into distinct groups (dashed red line). 2. Responses do not form distinct clusters and instead form a continuum. The response heat map as in 1 thus gives rise to one clear transition between On- and Off-type cells. In this case a hierarchical agglomerative clustering algorithm applied to a pairwise distance matrix representing the above heatmap results in a dendrogram (green) that is only divisible into two groups (dashed red line), each of which constitutes a continuum.

Action potential times were defined using a spike sorting application available in the Spike2 software package. Spike waveform templates were created using an appropriate threshold. Separate templates judged to belong to the same neuron were merged and templates indicative of noise were discarded though in the majority of cases this was not necessary as a single template was often constructed by the software (i.e. the variance in spike waveform was minimal whereas the signal to noise ratio was maximal) (Fig 1C). For each of the 4 chirp stimuli, handled separately, stimulation and response channels were segmented into 20 equally sized sections slightly off center of each chirp event, -0.4 seconds to 0.5 seconds from chirp onset (Fig 1B). Each of these 20 segments was further segmented in the following manner: “beat cycle 1” (0–0.2 sec) “beat cycle 2” (0.2–0.4 sec) occurring pre chirp onset and “Chirp window” (0.4–0.5), “beat cycle 3” (0.5–0.7 sec) and “beat cycle 4” (0.7–0.9 sec) occurring post chirp onset. Beat cycles (1–4) were then time coded such that each beat cycle would commence at 0 mV and initially be positive going in sign. Additional segmentations include a general “chirp centered” segment (0.3–0.5 sec) and more specific segmentations catered to the 4 stimuli separately with the goal of concatenating these segments across stimuli. Specifically catered segmentations included; (chirp 0) “0 phase” commencing at onset and ending at the second instance of phase π/2, (chirp π/2) “90 phase” commencing at onset and ending at the second instance of phase π, (chirp π) “180 phase” commencing at onset and ending at the second instance of phase 3π/2 and lastly (chirp 3π/2) “270 phase” commencing at onset and ending at the second instance of phase 2π. These segments were concatenated in order of increasing phase value to generate an abbreviated representation of responses to all chirps used in the current study that we will refer to as “All chirp phases”. Some of these initial segments were further combined at later stages of processing for various purposes of analysis. Peristimulus time histograms (PSTHs) of stimulus segments were generated by building a histogram from spike times, dividing the histogram values by its bin size (0.1 ms) to impose a time domain, multiplying this result by the number of trials under consideration and then smoothing with a 6 ms long box car filter. Smoothing artefacts due to filtering onset and offset were eliminated by triplicating the histogram and taking the central portion as the final PSTH.

### Pyramidal cell classification

On-type neurons possess basal dendrites that receive direct input from sensory afferents and respond to increases in EOD amplitude, whereas Off-type neurons lack basal dendrites and instead receive sensory afferent input indirectly through an inhibitory disynaptic relay and thus respond instead with increased firing rates during EOD amplitude decrease [26, 27]. In order to classify recordings into On- and Off-type we considered the phase of response to the 5 Hz beat component of our 4 small chirp stimuli. For this purpose we looked at the two beat cycles preceding chirp events (beat cycle 1 and 2 as defined above). We combined these two beat cycles from all 4 stimuli to evaluate responses based on a total of 32 seconds of a 5 Hz beat or a total of 160 trials. Phase preference was taken to be the phase of a synchrony vector known as vector strength, a measure commonly used to quantify the degree of phase locking exhibited by neurons when driven by a periodic stimulus [45]. This phase value was used to classify neurons as On-type (0< phase≤ π) or Off-type (π< phase≤2 π).

$$VS=\sqrt{{\left(\frac{1}{n}\sum_{i=1}^{n}cos\;\theta_{i}\right)}^{2}+{\left(\frac{1}{n}\overset{n}{\underset{i=1}{\sum }}sin\;\theta_{i}\right)}^{2}}$$

$$Phase=\{\begin{array}{cc} \text{arctan}\left(\frac{\sum_{i=1}^{n}sin\;\theta_{i}}{\sum_{i=1}^{n}cos\;\theta_{i}}\right) & if\;\frac{1}{n}\sum_{i=1}^{n}sin\;\theta_{i}\geq 0\\ 2\pi +\text{arctan}\left(\frac{\sum_{i=1}^{n}sin\;\theta_{i}}{\sum_{i=1}^{n}cos\;\theta_{i}}\right) & \text{otherwise} \end{array}$$

We assessed response significance by computing a Z-statistic associated with vector strength [45] defined as n *VS*^2^, where n is the number of action potentials in the recording. Only neurons with a Z-statistic >4 were used for subsequent analyses as we could not otherwise confidently assign all neurons their On- or Off-type labels. Under these criteria we were able to confidently ascribe a phase preference to 82% of our recordings.

On- and Off-type neurons were then further subdivided into superficial, intermediate and deep cell types based on their baseline (i.e. in the absence of stimulation) firing rates. This is because there is a strong negative correlation between baseline firing rate and dendritic morphology (S1 Fig) [29, 30]. Cells whose baseline firing rates were less than 15 Hz were labeled superficial, cells whose baseline firing rates were greater than 15 Hz and less than 30 Hz were labeled intermediate, and cells whose baseline firing rates were greater than 30 Hz were labeled deep. Such a classification scheme has been used previously in the literature in order to reveal important functional differences between the different anatomical classes [34, 35, 39, 46–50].

For a subset of cells, the depth of the recording electrode was recorded and plotted against each cell’s baseline firing rate (S2 Fig). There was no significant correlation between both quantities (r = -0.05, p = 0.8, n = 33). This is most likely due to the fact that the depth at which recordings are obtained will strongly depend on the recording electrode’s orientation relative to the animal’s dorso-ventral axis as well as it’s rostro-caudal and medio-lateral position [51] and that it is possible to record extracellularly from both soma and dendritic trees of ELL pyramidal cells [52]. Thus, although there is a clear negative correlation between the baseline firing rate and the location of the soma within the pyramidal cell layer [28], our results suggest that it is unlikely that the recording depth will provide additional useful evidence for anatomical cell class assignment.

Finally, it is important to note that our recordings were stationary. Indeed, baseline firing rate estimates obtained at several intervals throughout the recording in between stimulation were not different from one another for each cell (t-test, p>0.1, n = 90).

### Isolating responses to natural communication signals

To distinguish responses to chirps from responses to the underlying beat alone, we aligned the PSTH response of beat cycles 1 and 2 with the PSTH of our chirp centered stimuli (see pre-processing section) such that the pre-chip beat and the post-chirp beat of the chirp centered section will align with 2 separate copies of beat cycles 1 and 2 enabling a subtraction of the beat response from the response to the beat and the chirp. The maximum non-zero value that remains in a 25 ms time window following chirp onset is then taken as the response to the chirp. To compare anatomical classes we simply computed the average response values of individual cells within a given grouping.

### Multidimensional scaling

Multidimensional Scaling was used here simply for visualization purposes and achieved using the MATLAB function “cmdscale” (MathWorks, Natick, MA)

### Common factor analysis

Common Factor Analysis (CFA) was used in the present study to reduce dimensionality before applying standard agglomerative hierarchical clustering routines [53]. CFA differs from Principal Component Analysis (PCA), which is strictly a data transformation technique where the reduced dimensions are a linear sum of the observed variables and are arrived at through a decomposition of the total variance [54]. CFA is in fact a statistical model where the observed variables are instead a linear sum hidden factors that are obtained through a decomposition of the common variance shared among variables [55]. Furthermore, in CFA, these factors may be permitted to be either orthogonal or oblique relative to one another (i.e. non-correlated or correlated) [56], unlike the principal components from PCA. CFA thus provides a more accurate reflection of the true relations among variables and thus observations within the factor model.

Response features were first chosen in an attempt to fully capture the wide range of variation in response properties observed in the raw dataset. Table 1 lists a set of 18 response feature types that capture the variability in responses observed in recordings, identifies what stimulus segment (beat cycle or chirp window) the response feature is related to and what data type the feature is representative of (i.e, PSTH, cycle histogram or spike times). Seventeen of the features types used to generate our high dimensional representation space were repeated across all four stimuli whereas only one of the feature types was a composite measure that already took into account responses to all 4 stimuli. This totalled 69 features.

**Table 1:** Identifying response features suitable for building a factor model. 18 measures were chosen to capture the variation observed across the entire population of ELL pyramidal cell neurons in response to a communication signal (i.e. “small chirp”) occurring at different phases (0 90 180 and 270 degrees) of a continuous beat cycle. Measures are described and their origin within the complete stimulus waveform are indicated. Beat cycles (1–2) precede chirp onset while beat cycles (2–4) proceed the 100ms chirp window following chirp onset. The “data type” refers to the distinct stages of preprocessing from which the 18 measures originate (spike times, cycle histogram or PSTH). The total number of measures considered “Number per neuron”, and of those, the ones that are normally distributed are tallied. The identities of the stimuli (i.e. chirp phase or beat) from which the normally distributed measures belong are indicated. Collinear relationships among this subset of measures are identified. From the collinear pairs of measures identified one was randomly chosen for removal to yield the final features used in CFA classification which are similarly tallied.

'Measure #'	'Feature Descriptor'	'Stimulus Segment'	'Data type'	'Numberper Neuron'	'Normally Distributed'	'Stimulus Id'	'Collinearities'	'Final Features'
1	'ON OFF Index'	'Beat Cycle 1–4'	'Single PSTH'	1	1	'Across All Stimuli'	0	1
2	'Phase from Vector Strength'	'Beat Cycle 1–4'	'Cyclohistogram'	4	0	'none'	0	0
3	'Mean 1st Spike Latency'	'Beat Cycle 1–4'	'Spike Time'	4	4	'0 90 180 270'	0	4
4	'Std 1st Spike Latency'	'Beat Cycle 1–4'	'Spike Time'	4	4	'0 90 180 270'	0	4
5	'Mean last Spike Latency'	'Beat Cycle 1–4'	'Spike Time'	4	4	'0 90 180 270'	0	4
6	'Std last Spike Latency'	'Beat Cycle 1–4'	'Spike Time'	4	4	'0 90 180 270'	0	4
7	'Mean Spike Per Trial Count'	'Beat Cycle 1–4'	'Spike Time'	4	4	'0 90 180 270'	13	4
8	'STD Spike Per Trial Count'	'Beat Cycle 1–4'	'Spike Time'	4	2	'0 90'	0	2
9	'Mean Response Spike Time PSTH'	'Beat Cycle 1–4'	'Spike Time'	4	4	'0 90 180 270'	0	4
10	'Std Response Spike Time PSTH'	'Beat Cycle 1–4'	'Spike Time'	4	4	'0 90 180 270'	0	4
11	'Mean Response Spike Time Vector Strength'	'Beat Cycle 1–4'	'Spike Time'	4	0	'none'	0	0
12	'Std Response Spike Time Vector Strength'	'Beat Cycle 1–4'	'Spike Time'	4	0	'none'	0	0
13	'Mean PSTH value'	'Beat Cycle 1–4'	'Separate PSTHs'	4	4	'0 90 180 270'	7	0
14	'Std PSTH value'	'Beat Cycle 1–4'	'Separate PSTHs'	4	1	'0'	0	1
15	'Mean Response Spike Time'	'Chirp window'	'Spike Time'	4	3	'0 90 270'	0	3
16	'Std Response Spike Time'	'Chirp window'	'Spike Time'	4	1	'0'	0	1
17	'Mean 1st Spike Latency'	'Chirp window'	'Spike Time'	4	4	'0 90 180 270'	0	4
18	'Std 1st Spike Latency'	'Chirp window'	'Spike Time'	4	4	'0 90 180 270'	0	4
Number Of Response Features				69	48			44

The “On Off index” was computed by building a PSTH from all four Beat Cycles. Phase of the peak firing rate (θpfr) was used to center a window “A” ± π/2 with the remainder of the PSTH making up a second window “B”. If 0< θpfr ≤ π window “A” was defined as the “On” and window “B” defined as the “Off” window, if this was not the case then the opposite assignment applied.

$$ON\;OFF\;index=\frac{\text{On}-\text{Off}}{\text{On}+\text{Off}}$$

Phase from Vector strength was computed using beat cycles both prior to and following chirp onset. A number of measures were computed by combining the spike times from all four of these beat cycles, a total of 80 trials: mean and standard deviation were computed for first and last spike latency as well as for a per trial spike count. Response spikes were defined in two separate ways. Either a phase index corresponding to peak firing rate of the PSTH or a phase index obtained from vector strength was used to identify synchronous cross trial spiking events. The mean and standard deviation of these events where then computed. The last features considered of the beat response were the mean and standard deviation of all four beat cycle PSTHs (i.e. four PSTH being computed separately). Finally mean and standard deviation was computed for both first spike latency and the timing of response spikes within the chirp window commencing at chirp onset. In this case the response spike was relative to the peak firing rate of the PSTH. This tallied to a potential 69 features or dimensions to be reduced by our factor model. However, our criteria for inclusion in an effort to arrive at factor models that could successfully estimate the original correlation matrix was that features have a normal distribution and that no 2 variables possess glaring collinearities. The 48 features found to be normally distributed were thus further scrutinized as a group for collinearities. Collinearities were found between the “mean per trial spike count” taken from the four beat cycles (feature type 7) and the “mean PSTH values” also relating to the four beat cycles (feature type 13). The collinearities between these feature types were present for each of the four chirp stimuli so feature type 13 was not considered for inclusion in the factor model. Thus, an 8-factor model based on the remaining 44 features that permitted covariance among factors was generated. We found that the model was able to successfully estimate the reduced correlation matrix of the original variables (i.e. common variance) (χ^2^ = 1266, d.f. = 622, p = 3.47x10^-46^). Data was subsequently projected into the 8 dimensional coordinate space of the factor model where relative proximity between observations then reflects their similarity/difference. While the 8-factor model could already account for 78% of the total variance among the 44 variables, a 9-factor model added just 1% to the amount of total variance accounted for.

A one-sample Kolmogorov-Smirnov test (MathWorks, Natick, MA) was used as a test for standard normal distribution at a 5% significance level. Collinearities were identified by considering all pairwise comparisons between features and removing one variable from any pair whose distribution from unity was less than 0.02 standard deviations. After assessing features for inclusion an 8 Factor model was constructed (MathWorks, Natick, MA) utilizing the oblique “promax” rotation parameter.

### Dynamic time warping

The dynamic programming algorithm known as dynamic time warping (DTW) offers a very different approach from CFA in generating a representational space upon which to apply standard agglomerative hierarchical clustering routines. Widely known of in the speech recognition community DTW has additionally been applied to problems such as signature verification [57, 58]. This is possible since handwriting images can themselves be transformed into a time series. Due to its applicability to the more general problem of time series classification, the ability to represent neural responses here as time series (i.e. PSTHs), that there are fewer number of free parameters, no statistical assumptions to consider and more importantly the opportunity confirm results by applying two distinct algorithms, we here applied DTW to our data set. Unlike CFA which is a linear model where Euclidian among other distance measures are used as a proxy of similarity/dissimilarity between observations, DTW is a non-linear warping path across the time domain and a direct comparison between observations [59]. The distance between two observations within a factor model, or comparably the component space in PCA, is dependent on the composition of the population under consideration. An attractive feature of DTW is that warping distances between two observations are independent of all other observations but more importantly that non-linear relations among observations are preserved. The only restriction for DTW is that time series being compared be of equal length and the only one free parameter to consider is the degree of time warping permitted [60]. We used a script available through the MathWorks file exchange (mathworks.com/matlabcentral/fileexchange/43156-dynamic-time-warping—dtw-) which implements dynamic programing routines to compute the minimum warping path between two time series of equal length. We used a 25 ms warping window that corresponds to the integration time of ELL pyramidal neurons [47]. This window permitted the comparison of each index of one PSTH with those occurring at and up to 25 ms ahead in time of a second PSTH. It is important to note that this relationship is symmetrical so the direction of this comparison is irrelevant here. The cumulative distance computed as time series are compared culminates with the last computed value as the total length of the shortest warping path (a.k.a. the warping distance) between the two time series. PSTHs were generated from the “All chirp phases” summary/concatenation of responses to chirps. Sampling rate was decreased by 5-fold to reduce the computational time.

### Agglomerative hierarchical clustering

We applied an agglomerative hierarchical clustering algorithm to our data by first computing all pairwise comparison distance matrices from either the Euclidian distance within an 8 dimensional factor space or from the warping distance between PSTHs. Each of these matrices was then hierarchically linked based on the shortest distance between all observations contained within any two clusters. This was done using a single linkage agglomerative clustering algorithm (MathWorks, Natick, MA). For the purposes of visualizing dendrograms, structures were organized using the optimal leaf order function (MathWorks, Natick, MA). This function preserves the monotonic structure of the tree and finds the leaf arrangement that has the maximal amount of similarity among adjacent leaves. As such potential clusters are arranged adjacent to one another.

### Surrogate data

To test whether our data analysis techniques can actually detect the presence of clusters should they be present in our experimental data, we generated a surrogate dataset in the following way. First, the concatenated PSTH responses of six representative example cells from each type were chosen (i.e., one superficial, one intermediate, and one deep On-type, as well as one superficial, one intermediate, and one deep Off-type). Second, we reproduced the variability seen in our experimental data by computing the time varying standard deviation around the population-averaged PSTH response to all four chirps for each cell type. Independent and identically distributed low-pass filtered (Butterworth filter, 50 Hz cutoff) white noise processes with zero mean and the same time varying standard deviation were generated and each process was added to the template PSTH for each cell type with negative values of the firing rate set to zero. The 50 Hz cutoff frequency was chosen to mimic filtering by synapses [61]. The number of “cells” for each type was equal to that in our experimental dataset (i.e., we generated 18 superficial, 15 intermediate and 4 deep On-type surrogate responses, as well as 17 superficial, 14 intermediate, and 6 deep Off-type responses). This surrogate dataset thus consists of six response profiles to which variability mimicking that seen in the experimental data was added. Importantly, the surrogate dataset assumes that all heterogeneities in the response profiles within each cell type are due to addition of white noise. The surrogate dataset was analyzed using DTW followed by hierarchical clustering in the same way as our experimental data.

### Network projections

The first step to generating a network projection was to construct the network. For single link projections, adjacency matrices were constructed using a custom built code that operates on the output argument of the linkage function in MATLAB and determines which observations were linked at each node of the dendrogram. The n/2-NN network projection was constructed by connecting each observation with half of the data set for which that observation was closest based on Euclidian (CFA) or warping (DTW) distances. The values of adjacency matrices corresponded to the distance between observations (i.e. Euclidian or warping distance). All network projections were based on undirected graphs, created using the graph function (MathWorks, Natick, MA), and implemented the ‘force’ layout method, which applies attractive forces between adjacent nodes of the network and repulsive forces between distant nodes to achieve a particular projection in two dimensional space.

## Results

### Assessing responses of ELL pyramidal neuron within the lateral segment to natural communication stimuli

The aim of this study was to determine whether ELL pyramidal neurons could be functionally classified based solely on their responses to natural electro-communication stimuli and, if so, whether there was any correspondence between this functional classification and established anatomical classifications (Fig 1A and 1B). To do so, we recorded from ELL pyramidal cells in awake and behaving animals in response to natural stimuli (Fig 1C). One possibility (hypothesis A) is that pyramidal cell responses will form discrete clusters and can thus be functionally classified (Fig 1D, top). If this is the case, then there could be a correspondence between functionally and anatomically defined pyramidal cell classes. Alternatively (hypothesis B), pyramidal cell responses could instead form a continuum (Fig 1D, bottom). Should this be the case, it is possible that the responses of the different anatomical classes will occupy distinct regions within the continuum, which would indicate that there exists a relationship between anatomy and function. Alternatively, these could instead be scattered randomly throughout the continuum, indicating that there is no such relationship.

**Fig 2:**
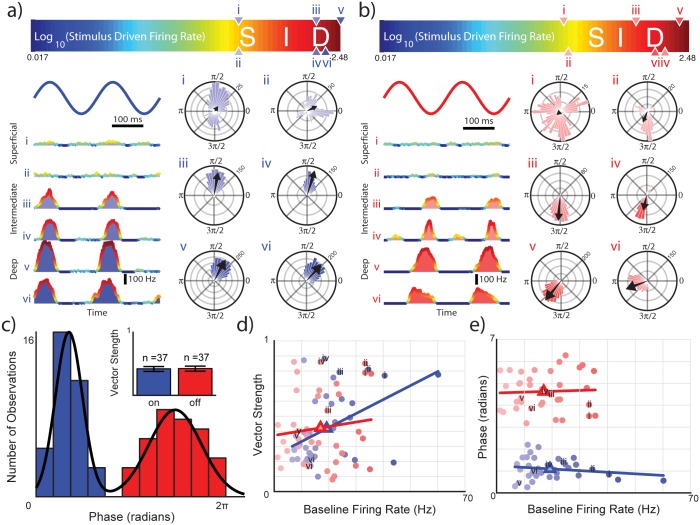
Responses of LS pyramidal cells to the beat. **A:** Peri-stimulus histograms (left) and cycle histograms (right) from six example On-type cells labeled according to phase of response to a 5Hz beat and baseline activity. Cells with higher baseline firing rates respond strongly to beats while those with lower baseline firing rates respond more weakly. Black arrows in the cycle histograms indicate the preferred phase and the length of the arrow gives the vector strength. Bin volume is indicated by values located at π/4 radians of each cycle histogram. Peak response magnitude values of example neurons are indicated by upward and downward pointing triangles on the colorbar (top) reflecting the logged stimulus driven firing rate. **B:** Same as in A but for six example Off-type neurons. **C:** Population distribution of response phase for all recordings in this study having a Z-stat ≥ 4. The histogram (bin size = π/6) reveals a bimodal distribution. Fitting the distribution with a Gaussian mixture model (black line) indicates an average on response at 1.08 radians and an average off response at 4.60 radians. The population (n = 74) is evenly divided into On- and Off-type neurons having mean vector strengths of 0.4175 ± SE 0.038 and 0.4226 ± SE 0.8664 respectively (panel inset). **D:** Linear regression models indicate a slight positive correlation of 0.445 exists between vector strength and baseline firing rate (p = 0.006) for On-type however no significant correlation exists for Off-type. **E:** No correlation exists between phase of response and baseline firing rate for either On-type or Off-type neurons as indicated by linear regression models. The rest is as in D.

We could easily distinguish between On- and Off-type neurons. Indeed, while On-type cells responded preferentially near the maximum (i.e., phase π/2) of the beat (Fig 2A), Off-type cells instead responded preferentially near the minimum (i.e., phase 3π/2) of the beat (Fig 2B). Plotting the distribution of the preferred phase across our dataset revealed a bimodal distribution (Hartigan’s dip test, *Dip* = 0.105, *p* = 0.001) with two well-separated modes (Fig 2C). On-type cells were assigned as belonging to the left mode (blue). This mode was centered at 1.08 radians had a kurtosis value near normality (k = 2.78) but was fairly positively skewed (s = 0.39). Off-type cells were assigned as belonging to the right mode (red). This mode was centered at 4.60 radians however had a lower value of kurtosis (k = 1.91) but was less skewed from normality (0.09). We also found a significant positive correlation between phase locking as measured by the vector strength and the baseline firing rate for On-type cells (PCC = 0.445, R^2^ = 0.1985, p = 0.0057, Fig 2D). In contrast, for Off-type cells, there was no significant correlation between vector strength and baseline firing rate (PCC = 0.124, R^2^ = 0.01534, p = 0.47, Fig 2D). We further found no significant correlation between the preferred phase and the baseline firing rate for either On- (PCC = -0.179, R^2^ = 0.03199, p = 0.29, Fig 2E) or Off- (PCC = 0.0467, R^2^ = 0.002178, p = 0.78, Fig 2E) type pyramidal cells. Overall, these results agree with previous ones [30, 41].

### Unsupervised classification of neural responses to naturalistic communication signals

We next applied 2 separate unsupervised classification algorithms to our dataset to test whether the responses of ELL pyramidal cells to chirp stimuli formed distinct clusters (i.e., Hypothesis A) or whether they form a continuum (i.e., Hypothesis B). These algorithms aim to uncover class structure by grouping similar objects together while keeping dissimilar objects separate (see Methods).

**Fig 3:**
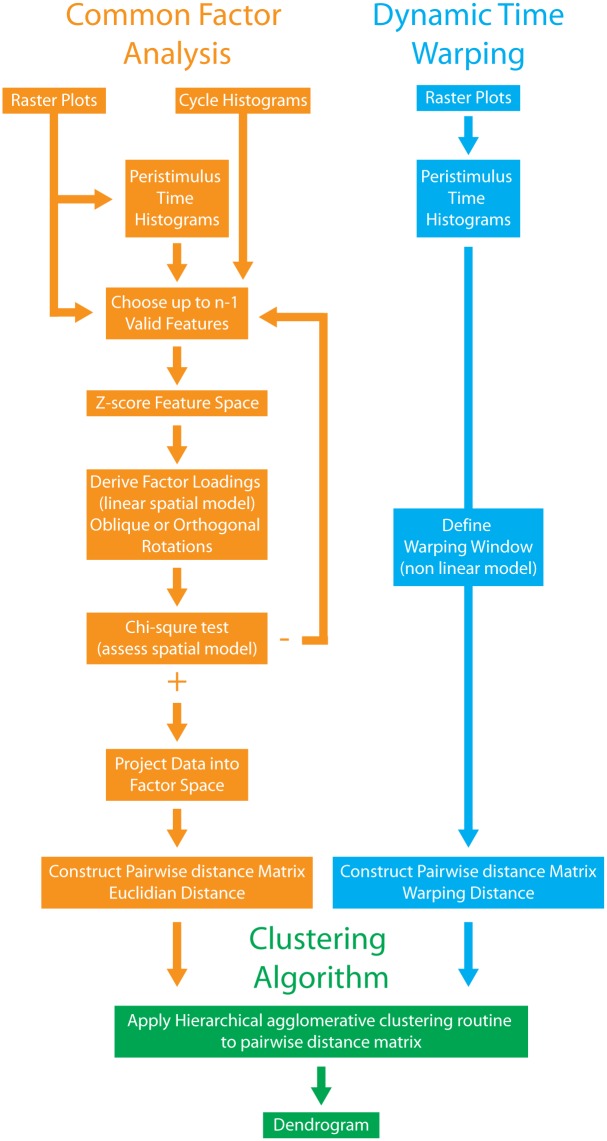
Summary of steps taken in order to classify neuronal responses to naturalistic communication stimuli. The Common factor analysis technique (orange) aims to reduce dimensionality by developing a linear statistical model summarising in a low dimensional space the high dimensional response space of the data. Proximities within this space can then be used determine how responses are represented in the brain (green) (i.e. discrete clustered representations or a continuous representation). In contrast, the Dynamic time warping technique (blue) permits one to directly quantify the proximity between observations via a non-linear relation among responses abstracted as time series. Raster plots are transformed into time series (PSTHs). After defining a window of comparison to be permitted between PSTHs all pairwise comparisons between observations belonging to a population are made, yielding a pairwise distance matrix, which can then be used in the same way as for the Common Factor Analysis technique (green).

The first algorithm quantified responses of each cell in our dataset by computing a large number of features (44, see Table 1) representing various aspects of the observed responses. The dimensionality of this set was then reduced by using a Common factor analysis (CFA) model (see Methods). It is important to note that CFA, like all dimensionality reduction algorithms, can only account for a portion of the variance displayed by the original dataset. In this case, we found that an 8-factor solution accounted for 78% of the variance. We then applied a single linkage agglomerative hierarchical clustering algorithm to the pairwise distance matrix constructed using the Euclidian distances between observations in the factor space (see Methods and Fig 3, left column).

In order to test that our results were not an artefact of choosing a large but finite feature space and of the limitations of the CFA dimensionality reduction algorithm, we also used a second unsupervised classification algorithm to functionally classify ELL pyramidal cells. Specifically, we used a dynamic time warping (DTW) algorithm, which is an elastic similarity method that allows for a non-linear comparison between a pair of time series (see Methods and Fig 3, right column). It is important to note that, as this methodology was applied on the PSTH responses of ELL pyramidal cells to natural communication stimuli themselves, DTW does not rely on dimensionality reduction of a finite feature space. A single-linkage agglomerative clustering algorithm was then applied to the pairwise distance matrix computed using DTW. The different steps used in both unsupervised classification algorithms are summarized in Fig 3.

### ELL pyramidal cell responses to chirps form a continuum

**Fig 4:**
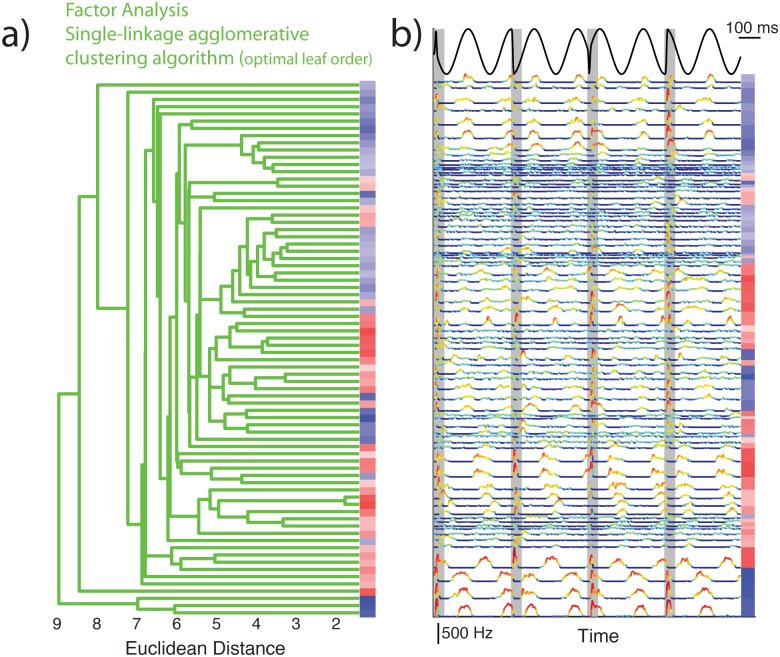
Pyramidal cell responses to chirp stimuli form a continuum based on an unsupervised classification algorithm including common factor analysis. **A:** Optimally sorted dendrogram (green) tracing the path of a single linkage agglomerative clustering algorithm from the leaves (right) to the root (left) as it was applied to the pairwise distance matrix representing Euclidian distance for all pairwise comparisons between observations projected into eight dimensions using a factor analytic model. The baseline firing rate is also indicated using the same color code as previously (see Fig 1A). **B:** Concatenated PSTH responses to the four chirp stimuli used in the study are presented for each neuron in the same order as the adjacent dendrogram. Responses are not normalized so that differences in response magnitude are readily apparent with the color gradient representing the logged stimulus driven firing rate allowing for a detailed visualization of each neuron’s stimulus preference. The baseline firing rate is also indicated using the same color code as previously.

**Fig 5:**
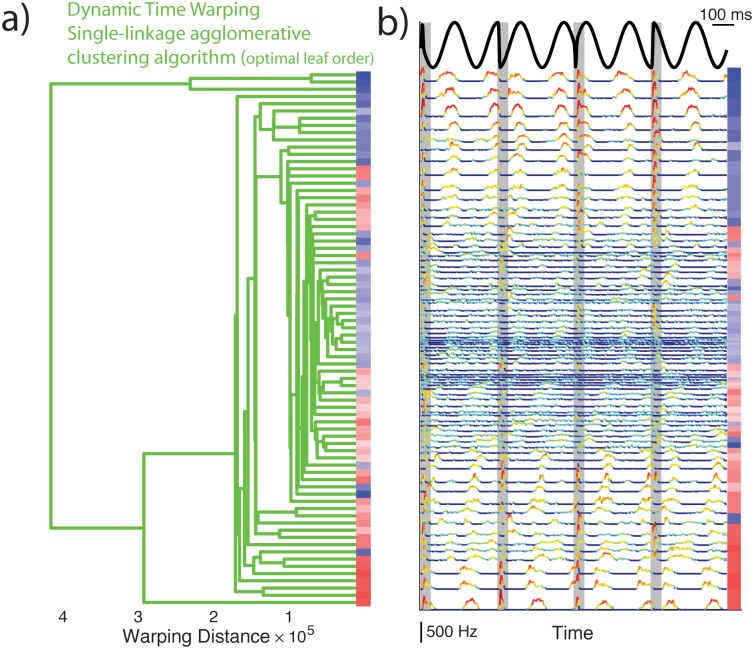
Pyramidal cell responses to chirp stimuli form a continuum based on an unsupervised classification algorithm including dynamic time warping. **A:** Optimally sorted dendrogram (green). The color code is the same as in Fig 4A. **B:** Summarized concatenated PSTH response to the four chirp stimuli used in the study are presented for each neuron in the same order as the adjacent dendrogram. The color code is the same as in Fig 4B.

Results obtained using the first and second unsupervised classification algorithms are shown in Figs 4 and 5, respectively, and were qualitatively similar. Figs 4A and 5A show the dendrograms with optimally sorted leaves such that the overall distance between observations on adjacent leaves is minimized. The baseline firing rate of each cell is furthermore color coded (blue for On-type and red for Off-type, darker hues indicate larger baseline firing rate values). For comparison, the PSTH responses of each cell are aligned with each dendrogram (Figs 4B and 5B). In both cases, it is seen that cells are sorted into blocks of adjacent On- or Off-type having similar baseline firing rates. Further inspection of PSTH responses reveals high similarity between adjacent responses and a grade change in response magnitude within blocks. Our results thus suggest that ELL pyramidal cell responses to chirp stimuli form a continuum (i.e., Hypothesis B).

### Relationship between function and anatomy in ELL pyramidal cells

So far, we have shown that ELL pyramidal cells cannot be functionally classified based on their responses to chirp stimuli alone. This is because applying unsupervised classification algorithms to the data showed that responses lie along a continuum rather than forming distinct clusters. In order to gain further understanding as to why this is the case, we now explore whether there is any relationship between the responses of the different anatomical ELL pyramidal cell classes.

To do so, we took advantage of the fact that there exists a strong correlation between the physiologically measured baseline firing rate and the anatomically measured apical dendritic length [29, 30] (see S1 Fig). Cells whose baseline firing rates were less than 15 Hz were labeled superficial, cells whose baseline firing rates were greater than 15 Hz but less than 30 Hz were labeled intermediate, and cells whose baseline firing rates were greater than 30 Hz were labeled deep. Such a classification scheme has been used previously in the literature in order to reveal important functional differences between the different anatomically defined classes [34, 35, 39, 46–50].

**Fig 6:**
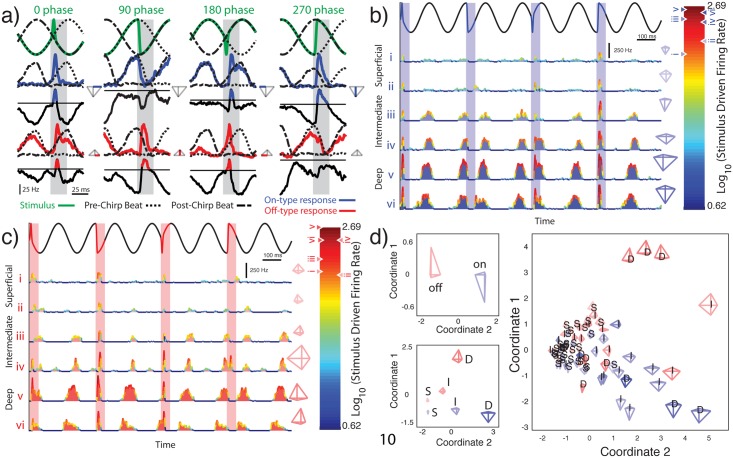
Responses of LS pyramidal cells to chirps. **A:** Illustration of the methodology used to differentiate between the responses to the beat and to the chirp. The chirp stimuli of interest are shown in green and the corresponding responses from typical On-type (blue) and Off-type (red) neurons are also shown running the full extent of the stimulus. The response to a beat stimulus is then aligned in phase with the beat of the stimulus of interest both before and after the chirp. These two alignments are indicated by two separate dashed lines identified as the pre-chirp and post-chirp beat and run the full extent of the stimulus of interest. Directly beneath actual responses is a signal which can take on both positive and negative values as it was generated by subtracting the pre-chirp and post-chirp responses from the response to the stimulus of interest. The line running though or above this signal indicates a value of zero with positive values highlighted an appropriate color. The maximum value of this signal within the grey window (25 msec after chirp onset) is taken as the response of the neuron to the chirp. Responses to each of the four chirps are used to generate a 2 dimensional representation of the 4 dimensional response space known as a glyph. The correspondence between glyph dimensions and neural response to chirp phases are demonstrated for average On- and Off -type examples. Correspondence is indicated by highlighting the glyph axis associated with a given chirp phase on the glyph seen to the right of that chirp phase response. **B:** Peri-stimulus histograms from the same six example On-type cells used in Fig 2. Responses to the 4 different chirps were concatenated. Note that, while responses of superficial On-type cells to the beat are difficult to discern from the PSTH’s in Fig 2, their responses to chirps are quite clear. A glyph summarizing each example neuron’s location within the response space to these four chirps is located to the right of their PSTH and their logged peak firing rate response is indicated by a leftward or rightward pointing triangle on the adjacent colorbar. **C:** Same as in B but for 6 example Off-type neurons. **D:** Representation of the response space to 4 natural communication signals averaging over different populations. (Top left) Chirp responses of all On-type cells were averaged along each dimension of response space to generate an average “On glyph”. The same was done for all Off-type cells. Multidimensional scaling was used to project the response space into two dimensions and glyphs where plotted centered on their two coordinate representation. The visualization procedure was repeated but for more specific subpopulations by dividing On- and Off-type further into deep, intermediate, and superficial (bottom left). For comparison, the glyphs from individual neurons are also shown (right).

Using this criterion, we found that our dataset was composed of 18 superficial, 15 intermediate and 4 deep On-type pyramidal cells and of 17 superficial, 14 intermediate, and 6 deep Off-type pyramidal cells. We then investigated how pyramidal cell heterogeneities influenced their responses to natural electro-communication “chirp” stimuli. To do so, we used four stimulus waveforms caused when a chirp occurs at different phases of the beat (Fig 6A, top panels, green). The responses of example On-type (blue) and Off-type (red) pyramidal cells to each chirp are shown in Fig 6A. Responses to all four chirps are then depicted as glyphs (Fig 6A).

The PSTH responses of different On-type cells to the different chirp stimulus waveforms are shown in Fig 6B. In general, On-type deep pyramidal cells responded more strongly to chirps than their intermediate and superficial counterparts and the strongest and weakest responses were elicited when the chirp occurred at phases 3π/2 and π/2, respectively (Fig 6B). Qualitatively similar results were seen for Off-type pyramidal cells: deep cells responded more strongly to chirps than their intermediate and superficial counterparts (Fig 6C). However, Off-type cells responded most strongly and weakly when the chirp occurred at phases π/2 and 3π/2, respectively, which is the opposite from what was observed for On-type cells (Fig 6C). As such, the responses of On- and Off-type pyramidal cells were significantly different from one another when considering chirps occurring at phases 3π/2 (One-way ANOVA, *p* = 0.0002) and π/2 (One-way ANOVA, *p* = 0.0003) but not at phases 0 (One-way ANOVA, *p* = 0.3518) and π (One-way ANOVA, *p* = 0.4107). Plotting the population-averaged responses of On- and Off-type cells to chirps revealed glyphs that were opposite of one another (Fig 6D, top left panel).

We further found that the responses of all 6 pyramidal cell classes were all significantly different from one another (*Wilks* = 0.46216, *F* = 3.9271, *p* = 0.00001, Fig 6D, bottom left panel), indicating that our classification scheme based on the baseline firing rate is unlikely to obscure any relationship between function and anatomy. However, we also found that there was considerable overlap between the responses of ELL pyramidal cells (Fig 6D, right panel).

**Fig 7:**
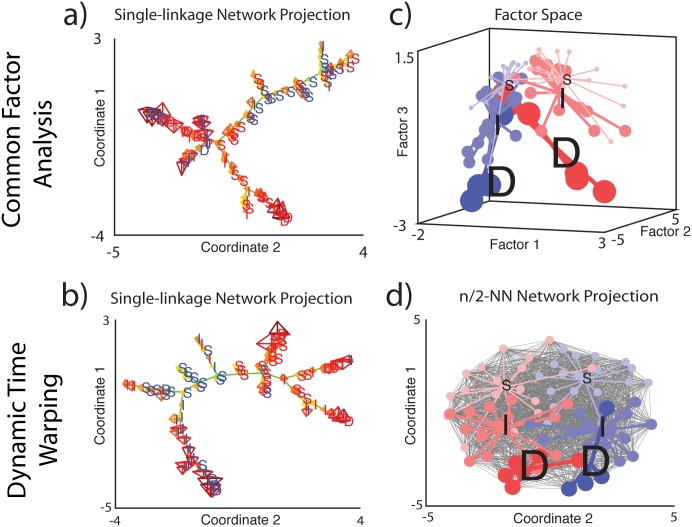
**A:** At each step of the single linkage algorithm clusters are merged on the basis of the minimum distance between two observations each belonging to separate clusters. Which observations were linked at each iteration of the algorithm (nodes of the above dendrogram) and at what distance they were from each other within the original pairwise distance matrix was used to generate an adjacency matrix or network that was then represented as a network graph. At each two dimensional coordinate the glyph summarizing the observations location within the response space to 4 chirp stimuli was plotted and a colored letter indicating On- (blue) or Off-type (red), deep (D), intermediate (I) or superficial (S) was plotted atop this. **B:** Same as A but for DTW. **C:** Response profiles captured by 44 response features projected into a three dimensional feature space using an 8 Factor statistical model (χ^2^ = 1266 d.f. = 622 p = 3.47x10^-46^) accounted for 78% of the variance. Each observation is colored according to its anatomical designation and each observation is connected to the mean value of its anatomical class within the factor space. **D:** Network graph (desaturated black lines) constructed from an adjacency matrix where each observation was connected to the closest one half of the population using the warping distance between observations as entries with each mode labeled according to On- or Off-type as well as anatomical cell class. The mean coordinate for each of the six anatomical designations was computed and observations were linked to their respective groups mean.

Our results so far suggest that there is a relationship between function and anatomy in ELL pyramidal cells that is somewhat blurred by large overlap between the responses of the different anatomical cell classes. In order to further test this possibility, we retraced the steps of our single linkage clustering algorithm used after either CFA or DTW and the results are shown in Fig 7A and 7B, respectively. Inspection of these 2D network projections reveals that On-type neurons tend to be located at one end while Off-type neurons tend to be located at the other end (Fig 7A and 7B).

If the 6 anatomical classes corresponded to 6 distinctive functional classes, then one could expect to see that observations grouped by anatomical class would correspond to 6 well separated non-overlapping clusters. Indeed, to test this hypothesis, we generated a surrogate dataset consisting of the PSTH responses of six examples cell from each anatomical class to which noise mimicking the variability seen in the experimental was added (see Methods). This surrogate dataset was then analyzed using DTW followed by hierarchical clustering. We found that the resulting dendrogram showed six well-separated clusters (S3 Fig). Labelling the individual “cells” from our surrogate dataset within a network constructed from the dynamic time warping algorithm revealed that the different anatomical classes occupy different quadrants (S4A Fig). This is however not the case for our experimental data. Instead, the different anatomical classes tend to be dispersed in all directions while occupying common quadrants. Confirming our previous observations, we did not find any clusters and the data were distributed in a rather uniform way along what might be described as two convergent planes each corresponding to On- or Off-type responses (Fig 7A and 7B). We next labeled individual neurons in either the factor space (Fig 7C) or within a network constructed from the dynamic time warping algorithm (Fig 7D). In both cases, we found that the representational space is highly meaningful as it relates to known anatomical and physiological properties (Fig 7C and 7D). Although there is some noise/overlap, on average the 6 anatomical classes occupy different regions that are themselves organized relative to one another in a meaningful way although there is clear overlap between adjacent regions, unlike what is seen for the surrogate dataset (S4B Fig). For example, superficial and deep classes are located at opposite ends of Factor 3 and the intermediate classes are located between these two while On- and Off-type neurons appear to be well separated along Factors 1 and 2, in agreement with the anatomical organization of ELL pyramidal cells (Fig 7C). Qualitatively similar results were obtained using dynamic time warping (Fig 7D). Overall, the results are in agreement with those obtained when considering response magnitude to the different chirps (Fig 6).

We conclude that, while the responses of the different anatomical cell classes are different from one another on average, considerable overlap between these implies that they are distributed along a continuum. On-type cells are located on one side and Off-type cells on the other. Furthermore, on each side, there is a general ordering of responses from deep to intermediate and from intermediate to superficial cells but it is the overlap between adjacent groups that prevents functional classification based on responses to chirp stimuli alone. Comparison between results obtained from our experimental and surrogate dataset suggest that this overlap is due to significant heterogeneity in responses within each anatomical class.

## Discussion

### Summary of results

We investigated ELL pyramidal cell responses to natural electro-communication stimuli. Specifically, we tested whether these responses could be used to functionally classify cells. Qualitatively similar results were obtained using two different unsupervised classification algorithms in that responses lied along a continuum. We furthermore investigated whether there was a relationship between anatomy and function. Overall, we found that the responses of On- and Off-type neurons could easily be distinguished from one another. The responses of superficial, intermediate, and deep pyramidal cells were different from one another on average, indicating that there is indeed a relationship between anatomy and function in ELL pyramidal cells. While the responses of different anatomical classes are ordered and occupy different regions in functional space, response heterogeneities within each anatomical class cause overlap between these regions, thereby forming a continuum. As such, our results provide strong evidence against the hypothesis that ELL pyramidal cells can be functionally classified based on their responses to natural electro-communication stimuli alone despite belonging to distinct anatomical classes.

### Potential caveats

Any functional classification is limited by the first step of data sampling [62]. It is conceivable limited sampling in general could have impacted the Factor model solution (i.e., CFA) derived from correlations among variables in the population. However, our results from the DTW time series classification do not suffer from this potential shortfall since, unlike CFA, the distances computed between each pair of neurons are independent of the population. Furthermore, DTW limits the number of free parameters to consider and allows for non-linear comparisons between neuron pairs. Given the good agreement between the two methods, as seen when comparing anatomical labeling in the factor space (i.e., CFA) and the n/2-NN network projection (i.e., DTW), as well as our results showing that clusters can be recovered from a surrogate dataset analyzed in the same way as the experimental data, it is unlikely that our results were an artefact of finite sampling or of our methodology.

There is a wide variety of clustering algorithms available including the commonly used k-means method. This method was not considered here due to a lack of spherically shaped clusters observed in the factor space [63]. Interestingly, the single linkage method used here has been criticised for producing chains [64], which could be argued to lead to the impression of a continuum. However, we note that all algorithms impose a structure on the data however the single linkage method is the only algorithm where the structure is least dependent on population composition. Essentially every observation is linked such that the minimum length of edges needed to connect every point in space is the final outcome of the algorithm. It is unlikely that our results were an artefact of using a single linkage algorithm as testing this algorithm on a toy data set with well-separated clusters chains were only observed locally within clusters and did not lead to the impression of a continuum. This is furthermore supported by results showing that using our methodology on surrogate data with the same variability as our experimental dataset but which consisted on six different response profiles by construction revealed six well-separated clusters.

We note that our anatomical class labels were assigned based on their baseline (i.e., in the absence of stimulation) properties rather than anatomical features per se. This is however unlikely to affect the qualitative nature of our results because: 1) previous studies have established a very strong linear correlation (-0.8) between morphology and baseline activity [29, 30]; 2) baseline activity is independent of stimulus driven activity and; 3) we found statistically significant differences between the average responses of On- and Off-type pyramidal cells as well as between the deep, intermediate, and superficial subclasses as determined using baseline activity. We note that, while it is possible to reconstruct ELL pyramidal cell morphology by filling the neuron with a tracer while recording intracellularly [29, 30], such methodology will not suffice in order to give unambiguous assignment. This is because measures of morphology such a dendritic length or spread are distributed along a continuum [29, 30]. Rather, one would need to label the cell being recorded from and the other five pyramidal cell types belonging to the same ELL column. Such techniques cannot be achieved in ELL pyramidal cells and are beyond the scope of the current study.

Finally, we note that our set of stimuli was limited to a beat as well as to four small chirp waveforms. It is conceivable that including responses to a greater stimulus set could lead to better separation between anatomical subclasses (i.e., deep, intermediate, and superficial). This is unlikely to be the case as pyramidal cells also display large heterogeneities in their responses to these stimuli [34, 46–48]. Our results showing that On- and Off-type cells could clearly be distinguished when only considering the beat (Fig 2C and 2E) but less so when adding the chirp stimuli (Fig 6D) supports our hypothesis but further studies are needed to test this prediction.

### Implications for coding in the electrosensory system

Our results show that the responses of different anatomical classes of ELL pyramidal cells were distributed along a continuum. An important question is then: why have different anatomical cell classes in the first place?

First, we note that deep pyramidal cells constitute a functionally separate population from their intermediate and superficial counterparts. This is because only deep pyramidal cells project to the nucleus praeminentialis (nP) [28, 29]. Neurons within nP in turn send feedback projections both directly and indirectly back to ELL pyramidal cells [65]. Previous studies have shown that deep pyramidal cells receive much less feedback than their superficial and intermediate counterparts and that, importantly, feedback to deep pyramidal cells is not plastic [29]. Thus, an important functional role for deep pyramidal cells is to provide feedback input primarily to their superficial and intermediate counterparts. Such feedback serves to attenuate responses to redundant stimuli [29, 47, 66–68] as well as provide gain control [69, 70].

Second, previous studies have compared responses of deep, intermediate, and superficial pyramidal cells to stimuli not considered in the current study including step increases in EOD amplitude, noisy time-varying waveforms, sinusoidal waveforms at different frequencies, other communication stimuli, and envelope stimuli [34, 41, 46–48]. In general, deep pyramidal cells showed the least selectivity in their response profiles that is reminiscent of that of peripheral afferents in general while superficial pyramidal cells showed the most selectivity. Some of this selectivity is due to feedback from deep pyramidal cells [29, 46, 47]. Interestingly, deep pyramidal cells tended to show more linear responses than their intermediate and superficial counterparts, which is in part due to their higher baseline firing rates [46, 71]. In general, it is thought that an important function of deep pyramidal cells is to provide the electrosensory brain with an accurate estimate of the actual stimulus independent of adaptation or filtering. This is because previous studies have shown that deep ELL pyramidal cells display little adaptation and have broad tuning curves when compared to their intermediate and superficial counterparts [28, 39, 47, 48, 72].

Our results showing that the responses of the different anatomical classes to natural electro-communication stimuli are significantly different from one another provide further evidence that there is a relationship between anatomical and functional classification of ELL pyramidal cells when considering their responses to natural electro-communication stimuli. However, this relationship is not one-to-one because response heterogeneities within each anatomical class cause overlap between responses of adjacent anatomical classes, thereby giving rise to a continuum. Despite these large response heterogeneities, we argue that it is important that future studies continue taking into account the different anatomical cell classes. This is because, as mentioned above, multiple studies including our own have found a relationship between anatomy and function in ELL pyramidal cells and because deep pyramidal cells constitute a distinct cell class in terms of anatomy and function.

We further argue that having ELL pyramidal cell responses to stimuli be distributed along a continuum provides more heterogeneity in their response profiles, which is in turn beneficial for coding. Indeed, both theoretical [73, 74] and experimental [34, 75, 76] studies have shown that neural heterogeneities are beneficial for coding. In particular, heterogeneities in ELL pyramidal cells are beneficial for estimating the characteristics of electro-communication stimuli used primarily during courtship behavior [34]. It is likely that such heterogeneities are beneficial for population coding of other types of electro-sensory stimuli but further studies are needed to test this hypothesis. This is because ELL pyramidal cells display correlations between their variability (i.e., noise correlations) [77, 78] that can only be estimated using simultaneous recordings. As such, population responses cannot be estimated by combining non-simultaneous single-unit recordings.

Moreover, the large diversity in responses afforded by a continuous representation is reminiscent of non-linear mixed selectivity, which is a well-known signature of high dimensional representations [79]. Neurons within the prefrontal and parietal cortices exhibiting such mixed selectivity are thought to behave as multitaskers by responding differentially depending on context, thereby performing different functions in different dynamically constructed ensembles [80]. Interestingly, since linear readout grows exponentially with dimensionality [80], such high dimensional representation would permit detection and/or discrimination of the diversity of stimulus features potentially encountered by the animal in its natural environment. We propose that heterogeneities in the responses of ELL pyramidal cells permit them to optimally encode behaviorally relevant stimulus features based on context. It is likely that the large amounts of neuromodulatory input received by ELL pyramidal cells help mediate this function [38, 81, 82].

We also argue that the strong heterogeneities in the response profiles of ELL pyramidal cells is beneficial for transmitting information to higher brain structures as all anatomical classes of ELL pyramidal cells project to the midbrain Torus semicircularis [29]. The responses of TS neurons are in general more selective than those of ELL pyramidal cells [32, 36, 71, 83, 84]. Interestingly however, some TS neurons show response profiles that are reminiscent of those of ELL pyramidal cells [36, 83]. It is conceivable that deep pyramidal cells project to such TS neurons while superficial and intermediate pyramidal cells instead project to more selective TS neurons but further studies are needed to test this prediction. It has been suggested that sparse selective responses of TS neurons serve to detect the occurrence of behaviorally relevant stimulus features while those of less selective TS neurons would instead serve to discriminate between different stimuli [36]. Both types of TS neurons furthermore project to higher brain areas [83]. We propose that the nonlinear mixed selectivity of ELL pyramidal cells is a mechanism permitting the emergence of selective and non-selective TS neurons.

## Supporting information

S1 Fig ELL pyramidal cells display strong correlations between anatomical and physiological properties. Plot of apical dendritic length as a function of baseline firing rate for On-type (blue) and Off-type (red) cells. The best-fit line is given by dendritic_length = 8613–145 × spontaneous rate (r = -0.73, p<10^−3^, n = 36). This figure is reproduced, with permission, from ref. [29].
(TIF)

S2 Fig No strong correlations were observed between recording depth and baseline firing rate. Plot of recording depth as a function of baseline firing rate. No significant correlation was found (r = -0.05, p = 0.8, n = 33).
(TIF)

S3 Fig Testing dynamic time warping and hierarchical clustering on a surrogate dataset consisting of six well-defined clusters. **A:** Optimally sorted dendrogram (green). The color code is the same as in Figs 4A and 5A. The red dashed line indicates a level at which the dendrogram can be partitioned to recover the six original clusters. **B:** Summarized concatenated PSTH responses to the four chirp stimuli used in the study are presented for each simulated neural response in the same order as the adjacent dendrogram. The color code is the same as in Figs 4B and 5B.
(TIF)

S4 Fig Network graphs obtained from surrogate reveal six well-defined clusters. **A:** Network graph obtained from the surrogate data. Glyphs summarizing the observations location within the response space to 4 chirp stimuli for each cell are also plotted. The colored letters indicate whether each cell was On- (blue) or Off-type (red) as well as either deep (D), intermediate (I) or superficial (S). Unlike the graph obtained from our experimental data (see Fig 7B), the cells are clearly ordered per type, allowing one to draw separating lines between these (dashed gray lines). **B:** Network graph from the surrogate dataset (desaturated black lines). Unlike the graph obtained from our experimental data (see Fig 7D), one can see six well-separated clusters.
(TIF)

S1 File This file contains the data used to generate the main figures in this manuscript. Please see “readme.txt” file for more information.
(ZIP)
