# Supplementary figures and images for: Electrosensory neural responses to natural electro-communication stimuli are distributed along a continuum
Source: PLoS One. 2017 Apr 6;12(4):e0175322. doi: 10.1371/journal.pone.0175322 (PMC5383285; doi:10.1371/journal.pone.0175322)

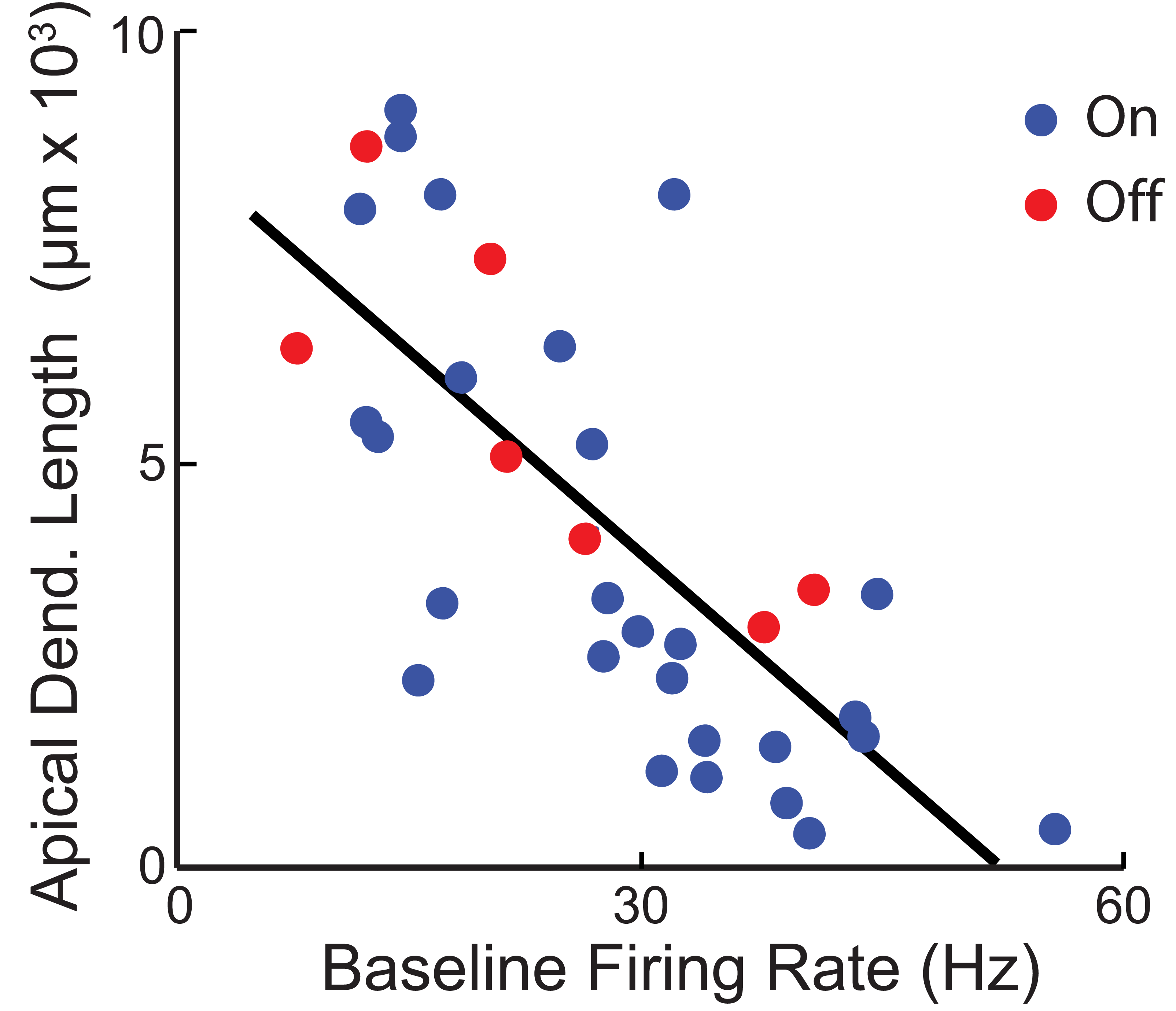

Supplement: S1 Fig — Plot of apical dendritic length as a function of baseline firing rate for On-type (blue) and Off-type (red) cells. The best-fit line is given by dendritic_length = 8613–145 × spontaneous rate (r = -0.73, p<10−3, n = 36). This figure is reproduced, with permission, from ref. [29]. (TIF) [file pone.0175322.s001.tif]

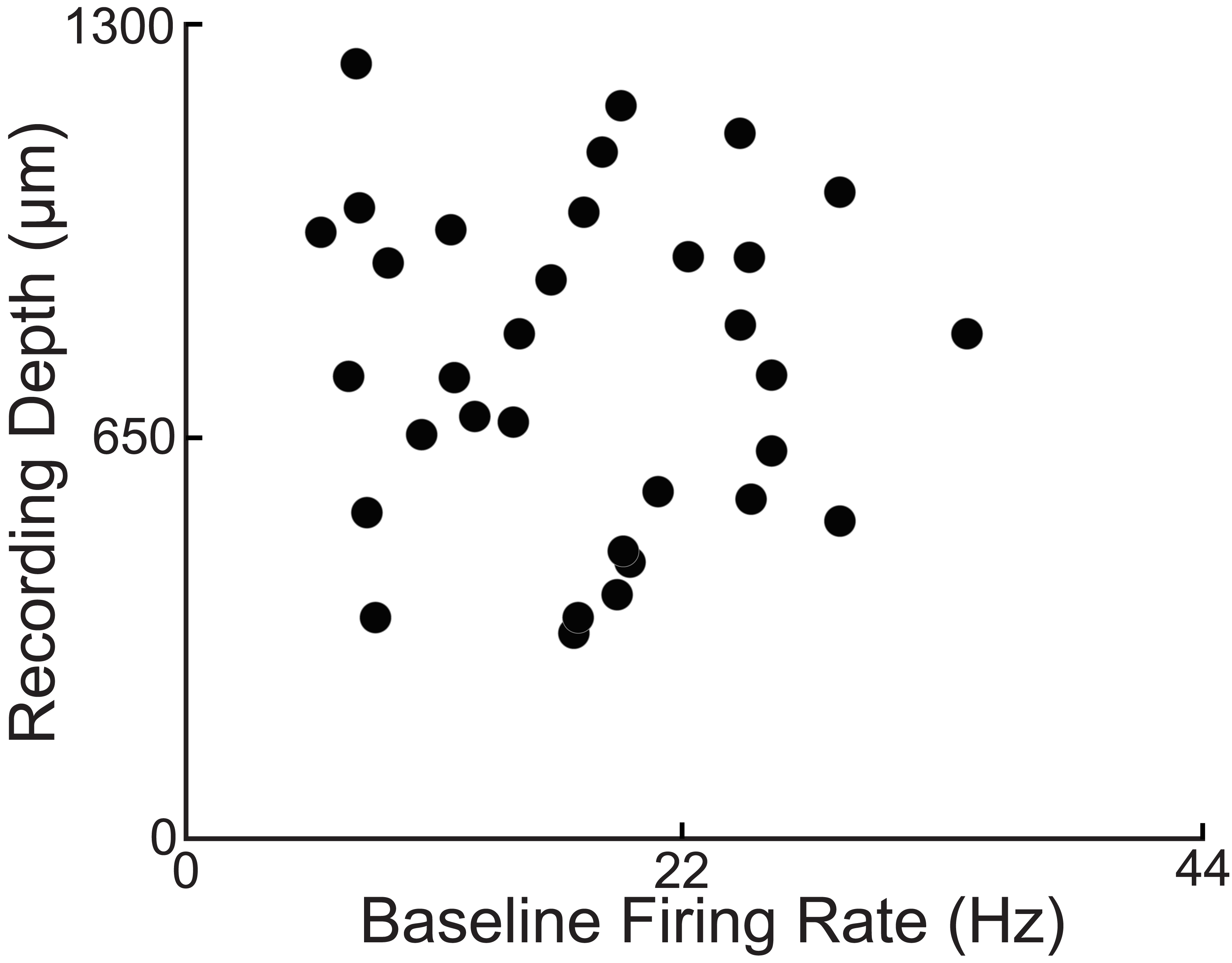

Supplement: S2 Fig — Plot of recording depth as a function of baseline firing rate. No significant correlation was found (r = -0.05, p = 0.8, n = 33). (TIF) [file pone.0175322.s002.tif]

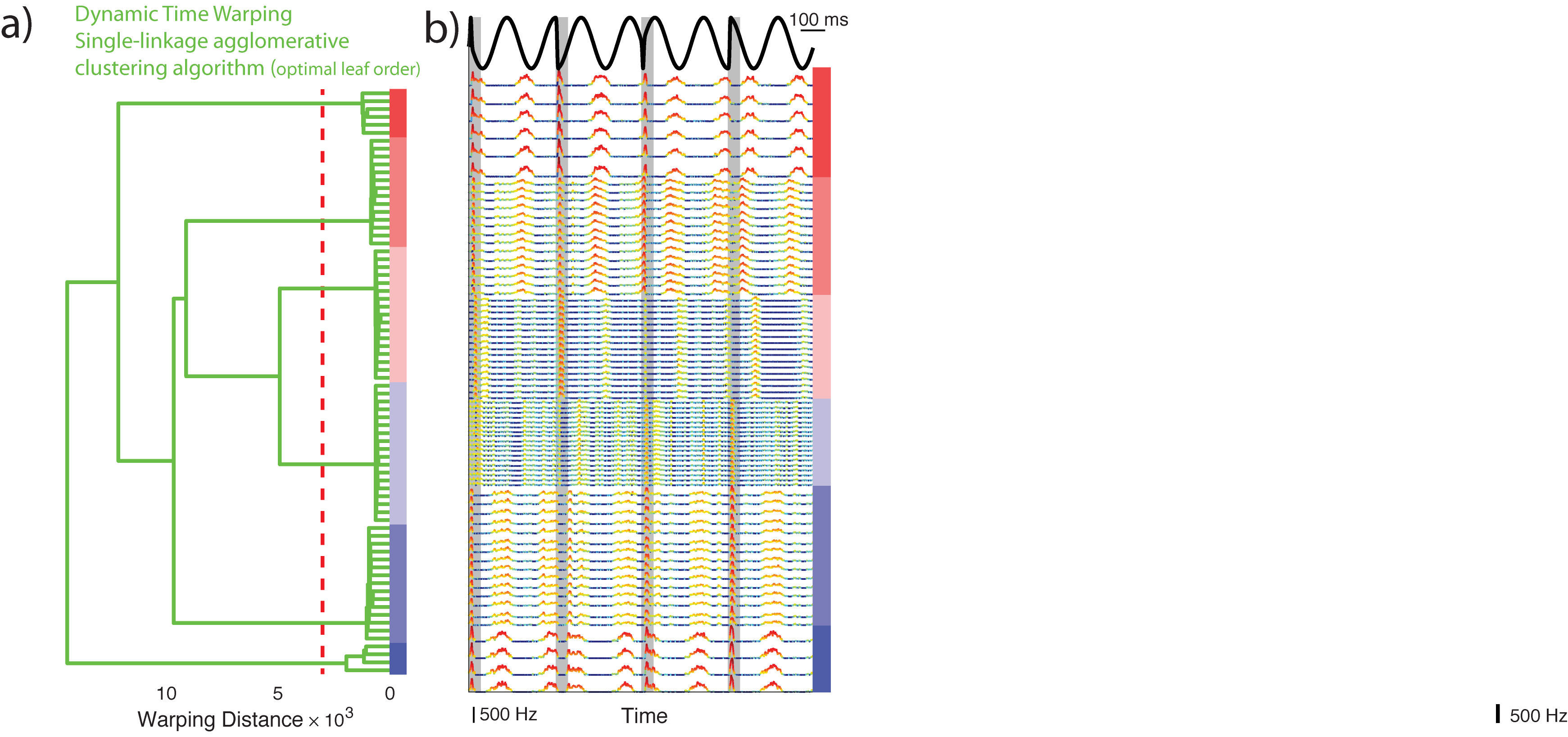

Supplement: S3 Fig — A: Optimally sorted dendrogram (green). The color code is the same as in Figs 4A and 5A. The red dashed line indicates a level at which the dendrogram can be partitioned to recover the six original clusters. B: Summarized concatenated PSTH responses to the four chirp stimuli used in the study are presented for each simulated neural response in the same order as the adjacent dendrogram. The color code is the same as in Figs 4B and 5B. (TIF) [file pone.0175322.s003.tif]

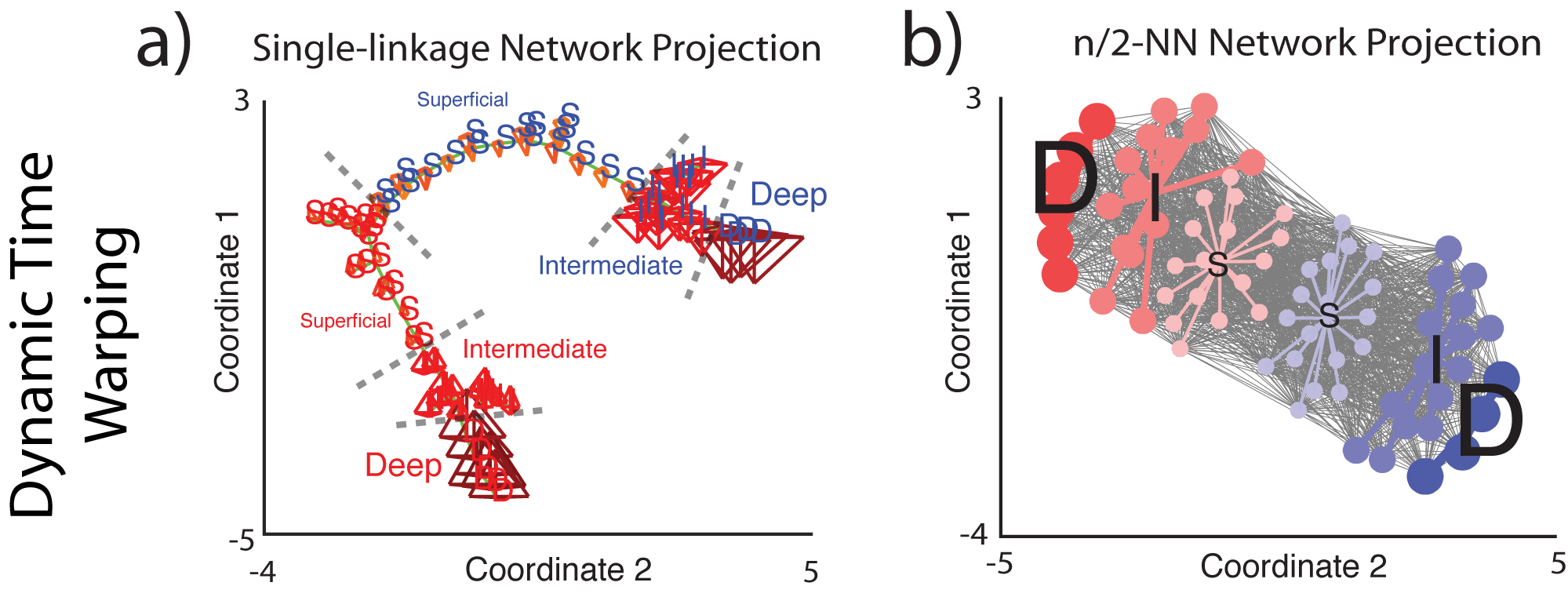

Supplement: S4 Fig — A: Network graph obtained from the surrogate data. Glyphs summarizing the observations location within the response space to 4 chirp stimuli for each cell are also plotted. The colored letters indicate whether each cell was On- (blue) or Off-type (red) as well as either deep (D), intermediate (I) or superficial (S). Unlike the graph obtained from our experimental data (see Fig 7B), the cells are clearly ordered per type, allowing one to draw separating lines between these (dashed gray lines). B: Network graph from the surrogate dataset (desaturated black lines). Unlike the graph obtained from our experimental data (see Fig 7D), one can see six well-separated clusters. (TIF) [file pone.0175322.s004.tif]
